# Neonatal mortality and associated factors in the specialized neonatal care unit Asmara, Eritrea

**DOI:** 10.1186/s12889-019-8118-x

**Published:** 2020-01-06

**Authors:** Amanuel Kidane Andegiorgish, Mihreteab Andemariam, Sabela Temesghen, Liya Ogbai, Zemichael Ogbe, Lingxia Zeng

**Affiliations:** 1 Department of Epidemiology and Biostatistics, School of Public Health, Xi’an Jiaotong University Health Science Center, No 76 West Yanta Road, Xi’an, 710061 Shaanxi Province People’s Republic of China; 2 Department of Epidemiology and Biostatistics, Asmara College of Health Sciences, School of Public Health, P.O.Box 8566, Asmara, Eritrea; 3 Department of Neonatology, Orotta School of Medicine and Health Sciences, Orotta National Referral Hospital, Asmara, Eritrea; 4 Key Laboratory of Environment and Genes Related to Diseases, Xi’an Jiaotong University, Ministry of Education, No 76 West Yanta Road, Xi’an, 710061 Shaanxi Province People’s Republic of China

**Keywords:** Neonatal mortality, Low birth weight, Associated factors, Specialized care unit, Eritrea

## Abstract

**Background:**

Limited knowledge on the magnitude of neonatal mortality and associated factors is hampering early intervention in African countries.

Objective: To determine neonatal mortality and associated factors in the Specialized Neonatal Care Unit Asmara, Eritrea.

**Methods:**

Medical records of all neonates admitted to the Specialized Neonatal Care Unit in 2016 were reviewed using a cross-sectional study. The most important causes of admission and mortality were analyzed. Univariate and multivariate logistic regression analysis was used to evaluate the strength of risk factors associated with neonatal mortality. Variables significant at *P* < 0.20 level in the univariate analysis were retained in the multivariate model. Model fit was evaluated using Hosmer and Lemeshow test (Chi-square = 12.89, df = 8; *P* = 0.116), implies the model’s estimates fit the data at an acceptable level. Collinearity was assessed using variance inflation factor (VIF) < 4. *P*-value < 0.05 was considered statistically significant.

**Results:**

Of the 1204 (59.9% boys and 40.1% girls) neonates admitted in 2016, 79 (65.6/1000 live births) died. The major causes of admission were sepsis (35.5%), respiratory distress syndrome (15.4%) and perinatal asphyxia (10%). Major causes of death were respiratory distress syndrome (48.1%); extremely low birth weight (40.9%) and very low birth weight (30.5%). After adjustment, low birth weight (Adjusted odds ratio (AOR) = 4.55, 95% CI,1.97–10.50), very low birth weight (AOR = 19.24, 95% CI, 5.80–63.78), late admission (24 h after diagnosis) (AOR = 2.96, 95% CI, 1.34–6.52), apgar score (in 1 min AOR = 2.28, 95% CI, 1.09–4.76, in 5 min AOR = 2.07, 95% CI, 1.02–4.22), and congenital abnormalities (AOR = 3.95, 95% CI, 1.59–9.85) were significantly associated with neonatal mortality. Neonates that stayed > 24 h in the Specialized Neonatal Care Unit (AOR = 0.23, 95% CI, 0.11–0.46) had a lower likelihood of death. Overall 95.8% of mothers of neonates attended antenatal care and 96.6% were facility delivered. None of the maternal conditions were associated with neonatal mortality in this study.

**Conclusions:**

Low birth weight, late admission, low apgar scores and congenital abnormalities were significantly associated with neonatal mortality in the Specialized Neonatal Care Unit. Early management of low birth weight, preterm births, and neonatal complications should be the priority issues for controlling local neonatal deaths.

## Background

Neonatal mortality, defined as death within the first 28 days of life, is a core indicator for neonatal health and wellbeing and is becoming a prominent component of overall under-five mortality. It is therefore receiving particular attention from Health authorities [1]. Even though the under-5 mortality rate dropped by 47% (from 9.9 million to 5.6 million children) from 2000 to 2016 globally, the neonatal mortality rate only fell by 39% over the same period [2]. Of the 5.9 million under five deaths reported in 2015, 2.7 million died during the neonatal period [3]. In 2013, sub-Saharan Africa contributed nearly half of the worlds under-5 mortality, mainly due to infectious diseases [4]. In 2017, UNICEF reported that neonatal mortality from sub-Saharan Africa and South Asia, accounted for 27/1000 live births. A child born from this region was nine times more likely to die in the first month of life than a child from a high-income country (https://data.unicef.org/topic/child-survival/neonatal-mortality/).

A number of previous studies have attempted to identify factors associated with neonatal admission [5, 6] and mortality [7–11]. Sepsis [6, 12], respiratory distress syndrome [9, 11–13], preterm birth [9, 12, 14–16], low birth weight [9, 12, 15–18], low apgars scores(a quantitative measure of neonatal vitality), at 1 and 5 min of birth [11, 12], low socioeconomic status [9, 10, 14, 19], cesarean section delivery [20], and neonatal age on admission [11, 14], are among the factors associated attributable factors of neonatal mortality [9–15, 19, 20].

Eritrea, with a population of 4.95 million has made tremendous advances in reducing neonatal mortality from 232/1000 in 1970 to 78.2/1000 in 2010 and 17.8/1000 in 2015 [21, 22]. The average annual reduction in under-five mortality rates from 1990 to 2013 in Eritrea was estimated to be 4.8% [23], whilst there was only 2.8% reduction for Africa and 2.9% for sub-Saharan Africa over the same period [23, 24]. The Eritrean Health Bulletin (special edition 2016) revealed that, the neonatal mortality rate declined by 47% between 1990 and 2015 (from 34/ 1000 in 1990 to 18/1000 in 2015) [23]. The control of infectious diseases, especially malaria, have been involved in this decline, since 70% of the population lives in malarious areas. However, the decline has not continued since 2015 and no change in neonatal mortality over the last 4 years has been reported in Eritrea [22].

Neonatal health, evaluated by the overall survival of neonates per live births, is a basic means of evaluating the overall health of a nation [19, 25]. Despite the fact that neonatal mortality rate in the specialized neonatal care unit (SNCU) in Asmara, Eritrea was lower than similar other neighboring countries [6, 11, 14, 26], neonates have still been dying, mainly from preventable causes. Implementations of effective neonatal care interventions are imperative for survival of neonates in developing countries [13, 15]. The framework of sustainable development goals by the year 2030 is to end preventable deaths and reduce neonatal mortality to less than 12 per 1000 live births [2]. Health system research on the evaluation of interventions and outcomes should be strengthened to complement this goal [10, 25, 27].

Therefore, this study was designed to evaluate neonatal mortality and associated factors among newborns admitted to the Specialized Neonatal Care Unit (SNCU) at Orotta National Referral Hospital (ONRH), Asmara, Eritrea in 2016.

## Methods

### Study design

A retrospective cross-sectional study was carried out to investigate neonatal mortality and associated factors among neonates admitted to the SNCU, Asmara, Eritrea.

### Study setting

ONRH is the nation’s tertiary hospital situated in Asmara, the capital city of Eritrea. The national referral hospital has different departments: Medical surgical, Gynocology-Obstetric (GynObs) and Pediatrics. Under the pediatric department there is a SNCU as detailed elsewhere [11].

The SNCU was established in 2003, through the collaboration of the Orotta teaching hospital, the Pediatric Association, and the Ministry of Health; with support from the Hummer Forum a German based Non-Governmental Organization. Later in 2013, other neonatal care units were established in four regional referral hospitals of the country.

The SNCU provides limited services on centralized oxygen supply, infusion pumps and incubators, radiant warmers and phototherapy equipment. It has no specialized nurses and ventilator machines, making it not qualified to provide care for preterm babies requiring the highest level of care.

### Study population

Information of all the 1414 neonates admitted to the SNCU from January1^st^ –December 31^st^, 2016 was retrieved and 1204 (85.1%) with complete information were included in the study. Neonates, either discharged immediately with advice, referred for further management or dead on arrival were excluded in accordance to the international classification of diseases (ICD-10).

### Data collection

Data was collected from four sources, i.e. the neonatal admission (log book), patient’s card, discharge form, and death registers. All information properly labeled by a neonatology specialist in register as per the ICD-10 standard was extracted using a structured checklist [11, 28]. Information on primary admission diagnosis (Sepsis, RDS, Perinatal asphyxia), gestational age (>= /< 37 weeks) and size appropriate for gestational age (AGA), small for gestational age (SGA) and large for gestational age (LGA), mode of delivery (spontaneous vaginal delivery (SVD)/cesarean section), place of delivery (gynecology-obstetric (GynObs), other facility/home), diagnosis to admission time(</> = 1 h), birth weight (normal birth weight (NBW), high birth weight (HBW), low birth weight (LBW), and very low birth weight (VLBW)), congenital anomality (yes/no), apgar scores (recorded either by the physician or primary care provider at the place of delivery), maternal age (years), obstetric complications (eclampsia/pre-eclampsia, diabetes mellitus), antenatal care visits, gravida and neonatal death (yes/no) was collected.

Small for gestational age (SGA), was defined as having a first weight recorded of less than the 10^th^ percentile and large for gestational age was defined as admission weight greater than 90^th^ percentile of weight-for-age and sex as defined by Intergrowth standards [28].

Data was collected by 3 public health graduates with previous experience. They were trained by the principal investigator and the neonatologist concerning the purpose of the study. The questionnaire was pretested on 20 neonatal cards of different admission years and modified according to the pre-test findings. Two hundred and ten (14.9%) of the initial 1414 neonates with incomplete information were excluded resulting in information on 1204 neonate’s admitted from 1^st^ of January to 31^st^ of December 2016.

### Statistical analysis

Double data input was performed using CSPro (version-7) software, and was exported to SPSS statistical package version 20 for windows (SPSS Inc., Chicago, IL, USA) for analysis. Quantitative indices are expressed as means ± standard deviations using t-test. Categorical variables are presented using frequency and percentage. Univariate and multivariate logistic regression analysis was performed to evaluate the strength of the relationship between neonatal death and risk factors. Variables that were significant at *P* value of < 0.20 level in the univariate analysis were included and retained in the multivariate model. We evaluated model fit through inspection of Hosmer and Lemeshow test (Chi-square = 12.89, df = 8; *P* = 0.116), which implies that the model’s estimates fit the data at an acceptable level. The potential presence of collinearity was assessed using variance inflation factor (VIF) < 4, and no collinearity was detected. Although *p* > 0.20 for gender and maternal conditions in the univariate analysis, we retained in the multivariate analysis based on the existing literature [11]. Furthermore, in the multivariate analysis, adjustment was made on neonatal and maternal conditions. Odds ratios (OR) and 95% confidence intervals (95% CIs) were calculated for each factor. Two-tailed *P* < 0.05 was considered statistically significant.

### Ethical considerations

Ethical approval was sought out from the Ministry of Health research ethics and protocol review committee in Asmara, Eritrea. Medical directors of ONRH, Pediatric department and the SNCU were briefed on the objectives of the study and written consent was obtained.

## Results

Ninety six percent of mothers of neonates had visited antenatal care during their pregnancy and 84.8% of the studied neonates were delivered in the tertiary GynObs unit of the hospital. Overall, 79.1% were delivered through spontaneous vaginal delivery and 20.9% through cesarean sections.

Ninety four percent were healthy mothers, while 3% had elevated blood pressure during pregnancy (eclampsia/preeclampsia). A total of 17 (1.7%) mothers had traditional practices for their neonates, such as uvilectomy, circumcision and initiation of additional foods (like butter) (Table 1).

**Table 1:** Demographic and Clinical Characteristics of neonates admitted to SNCU

Variables	Number	Percent (%)
Gender		
Male	721	59.9
Female	483	40.1
Gestation Age (Weeks)		
Full-term(> = 37 weeks)	962	79.9
Preterm(< 37 weeks)	242	20.1
Birth Weight (Kg)		
High birth weight (> = 4)	43	3.6
Normal birth weight (2.5–3.99)	766	64.0
Low birth weight (1.5–2.49)	305	25.5
Very Low birth weight (1.0–1.49)	59	5.0
Extremely Low birth weight (< 1.0)	22	1.9
Diagnosis to Admission time		
< 1 h	481	40.0
1 h −24 h	306	25.4
>= 24 h	417	34.6
Apgar Score (1 min)^a^		
Good Vitality(> = 7)	616	56.9
Poor Vitality(< 7)	466	43.1
Apgar Score (5 min) ^a^		
Good Vitality	866	79.8
Poor Vitality	219	20.2
Length of stay in SNCU		
< = 1 day	203	16.9
> 1 day	1001	83.1
Congenital Abnormality		
Yes	82	6.8
No	1122	93.2
Place of Delivery		
Gynecology-Obstetrics	1021	84.8
Other health facilities	142	11.8
Home Delivery	41	3.4
Mode of Delivery		
Spontaneous vaginal delivery	952	79.1
Elective CS	129	10.7
Emergency CS	123	10.2
Gestational Size		
Appropriate for Gestational Age	963	80.5
Small for Gestational Age	211	17.6
Lare for Gestational Age	23	1.9
Mother Age		
40+	68	5.6
30–39	408	33.9
20–29	679	56.4
< 20	49	4.1
Maternal Condition		
Normal	1130	93.9
Eclampsia/Pre-eclampsia	37	3.1
Diabetes Mellitus	9	0.7
Urinary Tract Infection	8	0.6
Others^b^	20	1.7
Antenatal Visit		
Yes	1154	95.8
No	50	4.2
Traditional Practice		
Circumcision	6	0.5
Uvilectomy	9	0.7
Additional Food	2	0.2
No traditional practice	1187	98.6

^a^N doesn’t include missing values

^b^Anemia, Cardiac disease, Febrile, Candidacies, HIV, and Epilepsy

### Causes of admission in to SNCU

As elsewhere, sepsis (culture proven or suspected) [11], was the major (35.5%) cause of admission, followed by RDS (15.4%), and perinatal asphyxia (10%). Thirty three percent of admitted neonates were LBW. The minimum recorded birth weight was 800 g. Congenital abnormality, including neural tube defect, digestive tract obstruction, anal-imperforation, congenital heart disease, and Downs syndrome, were among the commonly reported neonatal disorders 82 (6.8%). In addition, 95 (7.9%) neonates were admitted for observational follow-up (Table 2).

**Table 2:** Causes of admission in to SNCU

Causes of Admission	Frequency	Percent (%)
Sepsis	428	35.5
Respiratory distress syndrome	185	15.4
Perinatal asphyxia	120	10.0
For observation	95	7.9
Pneumonia	87	7.2
Congenital malformation	52	4.3
Jaundice	44	3.7
Hypernatrmic dehydration	38	3.2
Meconium aspiration syndrome	28	2.3
Hypothermia	26	2.2
Anemia	19	1.6
Gastrointestinal problem	18	1.5
Skin infection	17	1.4
Hypoglycemia	16	1.3
Trauma	12	1.0
Mastitis	9	0.7
Post circumcision / Uviloectomy bleeding	5	0.4
Renal problem	5	0.4
Total	1204	100.0

52 of the 82(63.4%) congenital malformation neonates were diagnosed on admission

### Causes of neonatal mortality

**Fig. 1:**
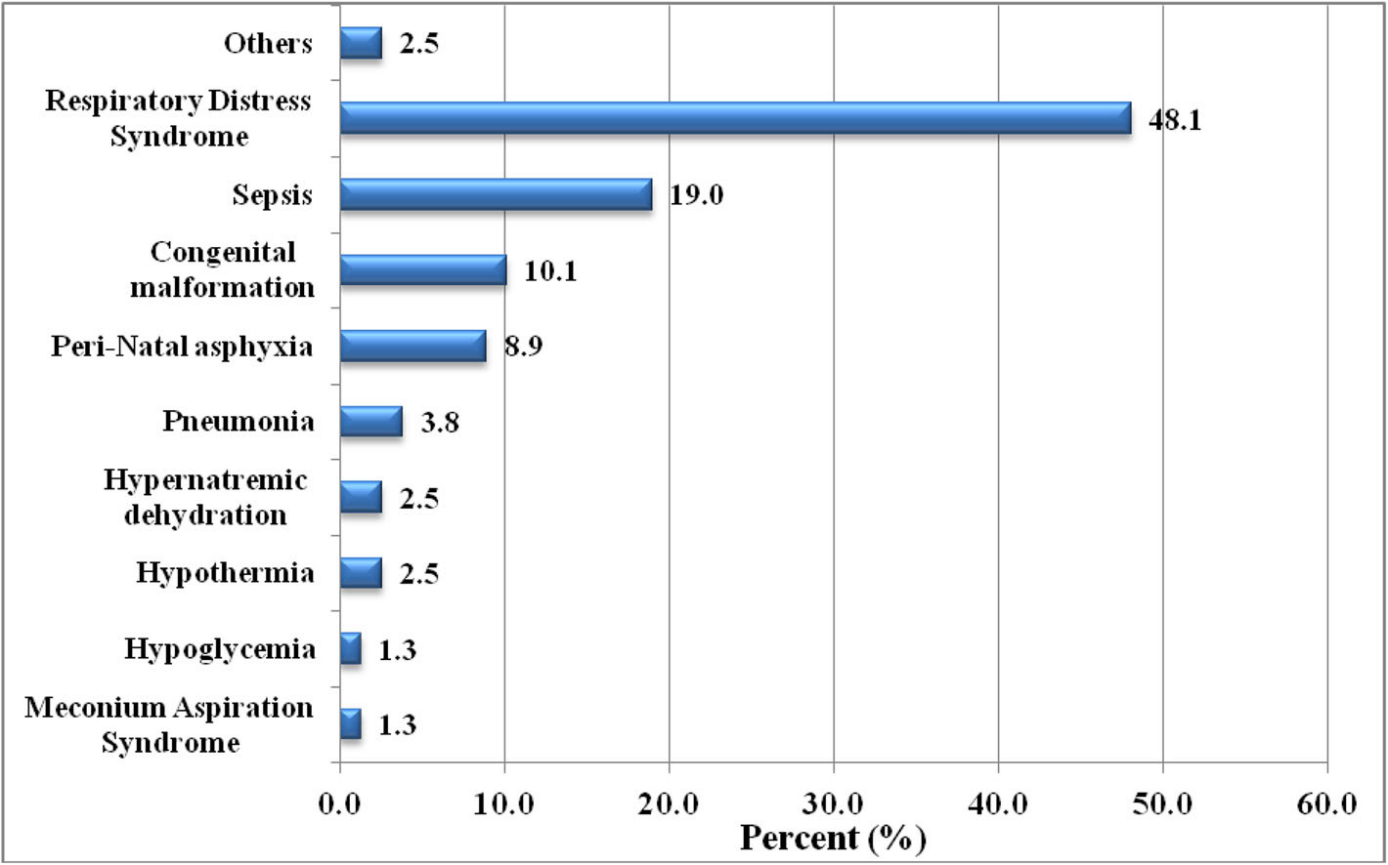
Cause of mortality in the SNCU. (**Others**: carbon dioxide poisoning and severe anemia)

Seventy-nine neonates died in 2016 in the SNCU. Preterm births with its complications were the major causes of neonatal mortality. RDS accounted for the highest (*n* = 38, 48.1%) cause of mortality followed by sepsis (*n* = 15, 19%) and congenital malformation (*n* = 8, 10.1%) (Fig. 1).

### Neonatal mortality and associated factors

Overall neonatal mortality in the SNCU was 65.6/1000 live births. There was a significant association between low gestational age (< 37 weeks), LBW (< 2500 g) and mortality (*P* < 0.001) (Table 3). The majority (40.9%) of extremely LBW (< 1000 g) neonates died.

**Table 3:** Univariate analysis of baseline neonatal and maternal variables to predict neonatal mortality

Variables	N	n (mortality/1000 live births)	OR & 95% CI	*P-value*
Gender				
Female	483	28(58)	1	
Male	721	51(71)	1.24(0.77–1.99)	0.381
Gestation Age (Weeks)				
Full-tem(> = 37 weeks)	962	39(41)	1	
Preterm (< 37 weeks)	242	40(165)	4.68(2.94–7.47)	< 0.001
Birth Weight (KG)^a^				
Normal birth weight	766	24(31)	1	
High birth weight	43	2(47)	1.51(0.35–6.60)	0.379
Low birth weight	305	25(82)	2.76(1.55–4.91)	< 0.001
Very low birth weight	59	18(305)	13.57(6.83–26.98)	< 0.001
Extremely low birth weight	22	9(409)	21.40(8.34–54.91)	< 0.001
Diagnosis to Admission time				
< 1 h	481	30(62)	1	
1 h-24 h	306	14(46)	0.72(0.38–1.38)	0.324
> =24 h	417	35(84)	1.38(0.83–2.29)	0.215
Apgar Score (1 min)^a^				
Good Vitality(> = 7)	616	17(28)	1	
Poor Vitality(< 7)	466	49(105)	2.65(1.37–5.11)	0.004
Apgar Score (5 min)^a^				
Good Vitality	866	34(39)	1	
Poor Vitality	219	32(146)	2.50(1.39–4.52)	0.002
Length of Stay in SNCU				
< = 1 day	203	20(99)	1	
> 1 day	1001	59(59)	1.75(1.03–2.97)	0.040
Congenital Abnormality				
No	1122	66(59)	1	
Yes	82	13(159)	3.01(1.59–5.73)	< 0.001
Place of Delivery				
Gynecology-Obstetrics	1021	59(58)	1	
Other Health Facility	142	16(113)	2.07(1.16–3.71)	0.014
Home Delivery	41	4(98)	1.76(0.61–5.11)	0.297
Mode of delivery				
SVD	952	70(74)	1	
Elective CS	129	4(31)	0.40(0.15–1.12)	0.082
Emergency CS	123	5(41)	0.53(0.21–1.35)	0.185
Gestational Size^a^				
Appropriate for Gestational Age	963	56(58)	1	
Small for Gestational Age	211	22(104)	1.89(1.12–3.16)	0.016
Maternal age				
20–29	679	39(57)	1	
< 20	49	7(143)	2.74 (1.15–6.48)	0.022
30–39	408	28(69)	1.21 (0.73–1.99)	0.458
> =40	68	5(74)	1.30(0.49–3.42)	0.592
Obstetric complication				
Normal	1130	73(65)	1	
Not normal	74	6(81)	1.28(0.54–3.04)	0.580
Antenatal care Visits				
yes	1154	76(66)	1	
No	36	3(83)	1.29(0.39–4.30)	0.678
Gravidity				
First	436	29(67)	1	
Multi	768	50(65)	0.98(0.61–1.57)	0.924

*n* neonatal mortality in each group, CS-Cesarean Section

^a^N does not include missing cases

Apgar score was > 7 for 61.2% and 79.8% in the first 1 and 5 min respectively. A significant inverse association was detected between Apgar score and neonatal mortality (*P* < 0.001) (Table 3).

Neonates with a congenital abnormality were also more likely to die (*P* < 0.001) as were length of stay (> 1 day) in the SNCU and place of delivery other than the tertiary GynObs (*P* < 0.05) (Table 3). However, mode of delivery, SVD (79.1%), elective cesarean section (10.7%), or emergency cesarean section (10.2%) and place of delivery (home vs health facility) were not associated with neonatal mortality (*P* > 0.05).

### Multivariate logistic regression analysis of neonatal and maternal variables association with neonatal mortality

Neonatal mortality among VLBW neonates was 19.24 times higher than normal birth weights, (AOR, 19.24, 95% CI: 5.80–63.78), and 4.55 times higher among LBW neonates (AOR, 4.55, 95% CI: 1.97–10.50) compared to NBW. Congenital abnormality neonates were 3.95 times more likely to die (AOR, 3.95, 95% CI: 1.59–9.85). Late admitted (after 24 h) neonates were almost three times more likely to die compared to their counterparts (AOR, 2.96, 95% CI:1.34–6.52). No association was found between maternal history of obstetric complications, multigravida and neonatal mortality. However, neonates of mothers who attended antenatal care were 72% less likely to die (Table 4).

**Table 4:** Multivariate logistic regression output for neonatal and maternal variables

Variables	B	S.E.	Wald	OR & 95% CI	*p*-value
Sex (Male vs Female)	0.27	0.30	0.82	1.32(0.73–2.38)	0.366
Birth weight					
HBW vs NBW	1.37	0.87	2.48	3.94(0.71–21.72)	0.115
LBW vs NBW	1.52	0.43	12.65	4.55(1.97–10.50)	< 0.001
VLBW vs NBW	2.96	0.61	23.38	19.24(5.80–63.78)	< 0.001
Gestational age(<37wks vs > =37wks)	0.38	0.41	0.84	1.46(0.65–3.26)	0.360
Diagnosis to Admission time					
1–24 h vs < 1 h	0.12	0.38	0.09	1.12(0.53–2.38)	0.759
> =24 h vs < 1 h	1.08	0.4	7.20	2.96(1.34–6.52)	0.007
Vitality1min(< 7 vs > =7)	0.82	0.38	4.83	2.28(1.09–4.76)	0.028
Vitality5min(< 7 vs > =7)	0.73	0.36	4.01	2.07(1.02–4.22)	0.045
Length of Stay in SNCU (> = 24 h vs < 24 h)	−1.48	0.36	16.6	0.23(0.11–0.46)	< 0.001
Congenital abnormality (yes vs no)	1.37	0.47	8.70	3.95(1.59–9.85)	0.003
Place of delivery					
other facility vs GynObs	0.70	0.48	2.15	2.01(0.79–5.11)	0.143
home vs GynObs	−1.18	1.29	0.83	0.31(0.02–3.87)	0.361
Mode of delivery					
Elective CS vs SVD	−0.78	0.64	1.50	0.46(0.13–1.60)	0.221
Emergency CS vs SVD	−0.01	0.56	0.00	0.99(0.33–2.95)	0.981
Gestational size (SGA/AGA)	−0.41	0.37	1.27	0.66(0.32–1.36)	0.260
Maternal age in years					
< 20 vs 20–29	0.27	0.62	0.19	1.32(0.39–4.46)	0.660
30–39 vs 20–29	−0.07	0.33	0.05	0.93(0.49–1.77)	0.825
> =40 vs 20–29	−0.83	0.79	1.11	0.44(0.09–2.05)	0.293
Obstetric Complications (Abnormal vs Normal)	0.06	0.57	0.01	1.07(0.35–3.24)	0.909
Antenatal care (no vs yes)	−1.29	0.96	1.78	0.28(0.04–1.83)	0.182
Gravida (Multi vs First)	0.17	0.33	0.26	1.18(0.62–2.27)	0.612

*CS* Cesarean Section

## Discussion

Overall, mortality among newborns admitted to the SNCU was 65.6/1000 live births. LBW and preterm births were the major associated factors of neonatal mortality, in which 66.6% of LBW and 50.6% of preterm neonates died. Previous studies concluded that these two factors were the direct and indirect causes of neonatal mortality [7–9, 14, 25, 26]. Preterm births are at high risk of RDS due to pulmonary surfactant deficiency in the lungs and are prone to hospital admission and death [12, 13, 29, 30]. Several interlinked factors could have resulted for this up surge in LBW babies, such as in-utero growth restriction associated with poor nutritional status of mothers of secondary to low socio-economic status [9, 10, 16–18].

Even though neonatal mortality in the SNCU was lower than similar settings of similar countries the causes of deaths remain the same [5, 6, 31]. Apgar score, a quantitative measure of neonatal vitality was significantly lower among deceased neonates both in 1 min (4.95 ± 1.88 vs 6.55 ± 1.73) and 5 min (6.26 ± 1.92 vs 7.80 ± 1.52), consistent with studies from Ethiopia and Kenya [5, 31], but different to a study from Tanzania [12].

Logistic regression analysis indicated that congenitally abnormal neonates were 3 times more likely to die than their counterparts, inline to many studies [12, 25, 29, 32], in which majority of neonates with congenital abnormalities died [7]. Management of congenital abnormality neonates requires advanced facilities and skilled care; which is not available in Eritrea.

Similar to other studies, neonatal admissions [5, 11, 30, 33, 34], and mortality were higher among boys than girls (64.6% vs 35.4%) [25, 34].

In common with other developing countries, sepsis, RDS and perinatal asphyxia accounted for more than 60% of admissions [5, 7, 8, 13, 25, 35]. In addition, after preterm births many studies have underlined that infections are the main cause of neonatal admission and death after the first week of life [8, 12, 14, 21, 36]. Infections, LBW and alleviation of an unhygienic environment should be targeted to maximize neonatal recovery [5, 35]. It is worth mentioning that pneumonia and sepsis, major causes of admissions in 2006, had declined by 66 and 50% respectively in this facility, and that the neonatal mortality rate had dropped by 18% despite the increased number of admissions compared to elsewhere [11]. This highlights that interventions on infection control, early diagnosis and treatment with improved hospital care are detrimental to neonatal survivals and must be further strengthened [12, 15, 25].

The majority (84.8%) of babies were delivered in the tertiary GynObs hospital, inline with previous studies [5, 6, 10, 11, 31]. The mortality rate was twice as high among other facility born neonates, but the difference was not statistically significant. Late admission, after 24 h of illness was significantly associated with mortality, which is similar to other findings from low resources countries [10, 12, 13], but contrary to an Ethiopian study [6]. This highlights that the outcome of early, skilled interventions in Eritrea is crucial for a variety of risks to newborn deaths.

SVD accounted 79% births of the studied neonates, which is in line to previous studies [5, 11], but different from a Kenyan study [31], which showed that nearly half of admitted neonates were delivered through cesarean section. Mothers preference on cesarean section is not common in Eritrea.

Neonatal deaths stem from poor maternal health, inadequate nutritional status [16–18], or care during pregnancy [9, 10, 14, 35]. Although no association was identified with maternal age, antenatal care visit, gravidity, and obstetric complications similar to other study [8], the high prevalence of neonatal mortality attributed to LBW may be a sign of poor maternal conditions [9, 10]. The results from this single SNCU study highlights the need for early identification and appropriate management of risks to reducing neonatal mortality and make the sustainable development goals achievable [2, 12, 27].

### Limitations

Using a cross-sectional study cause-effect relationship cannot be verified. Socioeconomic status (maternal education, household income and food security) for the most common associated risk factors of neonatal mortality in developing countries, especially in Africa, is not presented. Finally, our review was limited to documented cases and may under represent deaths of neonates born out of territory and during the first hospitalization, in which extreme low birth weight and congenital defects are at a higher risk of dying.

## Conclusion

Low birth weight, late admission, low apgar scores and congenital abnormalities were significantly associated with neonatal mortality in the Specialized Neonatal Care Unit of Orotta National Referral Hospital (ONRH), Asmara, Eritrea in 2016. Early management of low birth weight, preterm births, and neonatal complications should be the priority issues for controlling local neonatal deaths. Knowing factors related to specific contexts and designing interventions for the associated burdens is important.

## Data Availability

Pertinent data are presented in this manuscript. Additional data can be requested from the corresponding author upon reasonable request.

## Abbreviations

AOR: Adjusted Odd’s Ratio
APA: Appropriate for Gestational Age
CI: Confidence Interval
GynObs: Gynocology-Obstetric
LAG: Large for Gestational Age
LBW: Low Birth Weight
ONRH: Orotta National Referral Hospital
OR: Odd’s Ratio
RDS: Respiratory Distress Syndrome
SGA: Small for Gestational Age
SNCU: Specialized Neonatal Care Unite
